# How much salt do adults consume in climate vulnerable coastal Bangladesh?

**DOI:** 10.1186/1471-2458-14-584

**Published:** 2014-06-11

**Authors:** Sabrina Rasheed, Shamshad Jahan, Tamanna Sharmin, Shahidul Hoque, Masuma Akter Khanam, Mary Anne Land, Mohammad Iqbal, Syed Manzoor Ahmed Hanifi, Fatema Khatun, Abul Kasem Siddique, Abbas Bhuiya

**Affiliations:** 1 icddr,b, 68 Shaheed Tajuddin Ahmed Sarani, Mohakhali, Dhaka 1212, Bangladesh; 2 The George Institute for Global Health, Missenden Rd., PO Box M201, Camperdown, NSW 2050, Australia

**Keywords:** 24 h urinary excretion, Salt consumption, Coastal area, Climate change, Bangladesh

## Abstract

**Background:**

Evidence from numerous studies suggests that salt intake is an important determinant of elevated blood pressure. Robust data about salt consumption among adults in Bangladesh is sparse. However, much evidence suggests saline intrusion due to sea level rise as a result of climate change exposes more than 20 million people to adverse effects of salinity through the food and water supply. The objective of our study was to assess salt consumption among adults in a coastal region of Bangladesh.

**Methods:**

Our study was cross sectional and conducted during October-November 2011. A single 24 hour urine was collected from 400 randomly selected individuals over 18 years of age from Chakaria, a rural, coastal area in Southeastern Bangladesh. Logistic regression was conducted to identify the determinants of high salt consumption.

**Results:**

The mean urinary sodium excretion was 115 mmol/d (6.8 g salt). Based on logistic regression using two different cutoff points (IOM and WHO), housewives and those living in the coastal area had a significantly higher probability of high salt intake compared with people who were engaged in labour-intensive occupations and who lived in hilly areas.

**Conclusion:**

It is important to create awareness about the implication of excessive salt intake on health and to develop strategies for reducing salt intake that can be implemented at the community-level. A sustainable policy for salt reduction in the Bangladeshi diet should be formulated with special emphasis on coastal areas.

## Background

Hypertension is a major risk factor for cardiovascular diseases which now account for more than 27 % of all deaths in Bangladesh [1]. The amount of dietary salt consumed is an important determinant of blood pressure levels and a modest reduction in salt has been found to have a significant and, from a population perspective, important effect on lowering blood pressure [2-5]. It has been estimated that decreasing population-level salt intake from the estimated global levels of 9-12 g/d [6] to the recommended level of 5 g/d [7] would result in significant reduction of blood pressure and would reduce the world wide stroke rate and cardiovascular disease rate by 23% and 17%, respectively [8,9]. Achieving this reduction would project to prevent 2.5 million deaths worldwide each year [8,9]. While sodium is essential to sustain human life, the recommended daily consumption for meeting physiological need is only 1.5 g (3.8 g salt) [10]. The WHO recommendation of 5 g salt/d reflects a pragmatic compromise between the beneficial and achievable in terms of reducing salt consumption [7].

No current definitive estimate of population salt consumption in Bangladesh exists. Of two reported studies, one involves pregnant women in the southeastern coastal area and showed urinary sodium excretion of 170 mmol/day (equivalent 9 g salt) [11]. The other study was of 10 expatriate Bangladeshi patients with renal disease living in East London and showed that the two daily major meals combined to contain 10 g of salt per day [12]. The latter study is useful for understanding that a lot of salt is added to the Bangladeshi meal.

The low lying coastal belt of Bangladesh is highly vulnerable to the effects of climate change. Sea levels are rising, storms and cyclones are occurring more frequently, and soil and water salinity are increasing [13]. It is estimated that 20 million people living in coastal Bangladesh are already facing increased exposure to diseases like hypertension by the increased salinity of the water supply [14,15]. In coastal areas it is estimated that salt intake from drinking water can range from 1.2-16 g/day depending on the water source and the season [15]. Therefore, it is important that levels of salt consumption among adults in coastal areas are established and factors associated with high salt consumption are investigated.

## Method

### Study area

The study was conducted in Chakaria -a rural area of the Southeastern coastal region of Bangladesh - in October and November 2011. Locally produced raw salt is ubiquitous, widely used and inexpensive in Chakaria. Because of the area’s propensity for seasonal flooding and extensive shrimp cultivation, saline intrusion in the cultivable land has had a negative impact on local agriculture and livestock in some communities. Details of the study area are reported elsewhere [16]. For our study 3 unions (Bangladesh’s smallest administrative unit) were selected to represent geographic variation (plains, hilly and coastal). Village residents above 18 years of age were randomly selected for a 24 hour urine collection, anthropometric measurements and administration of a survey.

### Study subjects

Random sampling was carried out by selecting individuals from the existing Health and Demographic Surveillance System (HDSS), Chakaria. The list provided names and addresses of 3,581 adults over 18 years of age from 5 villages of 3 Unions representing geographical variations present in the area. The villages of the plains were large; therefore, two villages from coastal and hilly areas were randomly chosen to obtain a population pool similar to that of the single selected plains village.

**Figure 1:**
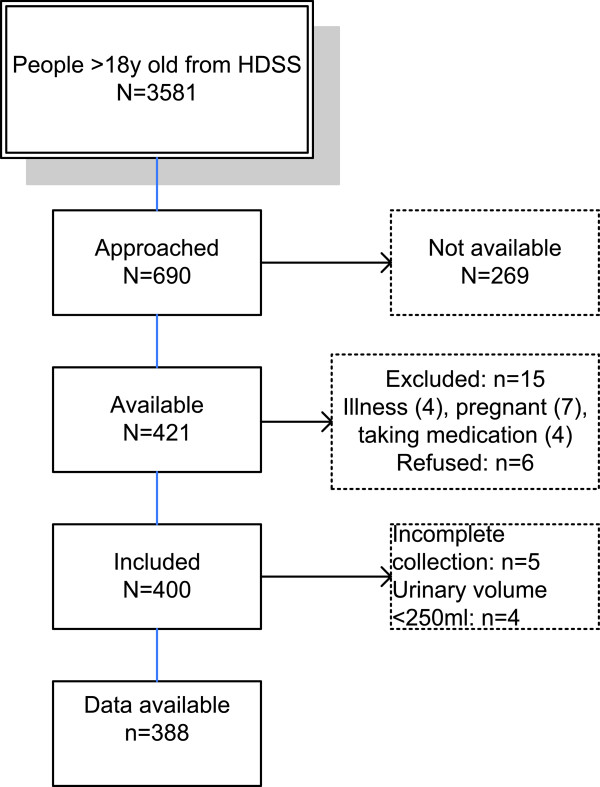
Recruitment and inclusion of study participants.

To calculate the required sample size for the study we used the following formula:

$$\begin{array}{l} n=\frac{t^{2}\mathrm{xp}\left(1-p\right)}{m^{2}}\\ \; \end{array}$$

n = required sample size, t = confidence level at 95% (standard value of 1.96), p = estimated prevalence of people consuming <5 g of salt/day (0.5), m = margin of error at 5% (standard value of 0.05) [17]. With the assumption that 50% of people will be consuming over 5 g of salt per day and a participation rate of 95% we invited 403 individuals to participate. From the list of 3,581 we invited every 3rd individual to participate in the study. If the selected person was not available the next person on the list was approached (Figure 1). Among those who were available 15 were excluded. A few people refused to participate (n = 6). Urine samples of 9 people were discarded due to suspected incomplete collection as determined by communication with the participant and urinary volumes < 250 ml. Complete data from 388 individuals were available for analysis. All selected participants were living independently in their own homes and reported no known illness.

All subjects provided written consent to participate. The study received ethical clearance from International Centre for Diarrheal Disease Research, Bangladesh (ICDDR, B).

### Recording of health variables

The weight of each subject was measured using a digital electric balance (Seca alpha, GmBH & Company, Range 0.1-150 kg); height was measured using a locally constructed scale. For both measurements the subject was instructed to be barefoot and to remove any heavy clothing. Body Mass Index was calculated from body weight and height measurements.

Three blood pressure measurement were taken from the right arm of the seated participant following a 5 minute rest period using OMRON HEM-90XL automated sphygmomanometer which is a valid method used in clinical settings. The first measurement was discarded and the mean of the second and third measurements was used in the analysis. All readings were taken by a trained data collector with at least a bachelors’ degree. Data collectors received a 2-day training on collecting anthropometric and blood pressure measurements. The training continued until measurements of the data collectors were ± 10% of the trainer. No measurement of inter-observer reliability was conducted in the study. In-field quality control was done through duplicate data collection for 10% of the respondents by field supervisors.

### 24 hour urine collection

The measurement of 24 hour urinary sodium excretion is considered the preferred method for determining population sodium intake [18]. The advantage of using this method is that it is not affected by bias related to self-reporting. However, it does not take into account electrolyte loss other than via kidney (such as sweat) and tends to underestimate true intake by 10-15% [18].

For our study 24 hour urine was collected in a clean plastic container which was given to the subjects along with detailed verbal instructions. A smaller plastic container and a bag were also provided so that people could collect the urine if they had to go outside of the house for short times during the 24 hour urine collection period. During collection of the urine sample questions were asked to determine the start and end time of the collection period and completeness of collection. The container was brought to the Chakaria field office where the urine volume was measured and 10 ml of each sample were preserved at -4°C for analysis. The 10 ml samples were transported to Dhaka for laboratory analysis to determine sodium and potassium levels in the urine.

To guard against under collection, urine samples were rejected if urinary volume was <250 ml. The individual sodium excretion values were the product of sodium concentration in the urine and urinary volume corrected to 24 hour and expressed in mmol/d. A factor of 22.99 was used to convert mmol to mg of sodium. For the conversion from g sodium to sodium chloride, a factor of 2.54 was used [19].

### Socioeconomic variables

The asset quintile was calculated based on ownership of 14 assets (almirah, table/chair, van/rickshaw, choki/khat or bed, radio, television, bicycle, motorcycle, fridge, sofa, electric fan, sewing machine, telephone and electricity) collected from the HDSS data. Principal component analysis was conducted for calculating weights of the asset index scores [20]. The scores were divided into 3 tertiles with the lowest tertile representing the poorest households and the highest tertile representing the richest. Data on age, education and occupation of selected individuals was also collected from the HDSS.

### Statistical analysis

Means, standard deviations and ranges of all the variables were calculated and normality of the data was checked. The proportion of people with high salt consumption was calculated using the cut off points (1.5 g of sodium) proposed by the Institute of Medicine (IOM) and (1.96 g of sodium or 5 g of salt) the WHO [10,18]. For univariate analysis, a Chi square test was used to the find difference between proportions. Two different logistic regression models were run using the 2 different cut off points as we were interested in the correlates of high salt intake in the population. The independent variables in the model included age, sex, occupation, education, household wealth index and geographic location of the subject’s residence. First, logistic regression with stepwise function was used to come up with 2 parsimonious models where all the variables significantly related to the outcome variable at the 10% level were included. Finally, variables that were included in both models (one with the IOM cut off, the other with the WHO cut off) were used to create the final models. A simple logistic regression was run to estimate the regression coefficients. All data were analyzed using SPSS version 17.

## Results

**Figure 2:**
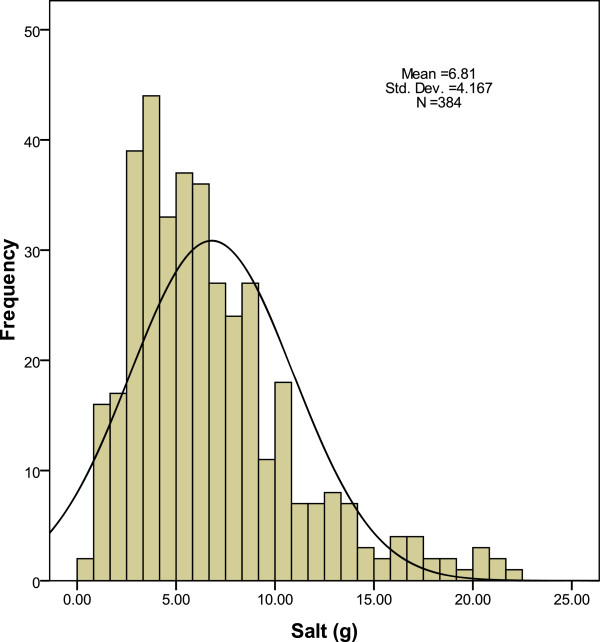
Distribution of salt excretion in gram/day.

The mean daily sodium excretion of the population was 115 mmol (equivalent 6.7 g of salt) per day. The mean total urinary volume was 1.5 litres (SD 0.79). The distribution of salt excretion was skewed towards the right with 59.6% of the population having a daily intake higher than the WHO recommendation (5 g of salt/day); 75% had a daily intake higher than the IOM recommendation (1.5 g of sodium/day) (Figure 2).

The mean age of the study participants was 44.6 years, with the range 25–105 years. The sample had a similar proportion of males and females. The majority of females were housewives whereas males were mainly in engaged in labour-intensive occupations. The population was distributed among hilly, coastal and plains which represented the geographical variation of the study area (Table 1). In bivariate analysis using both cut offs (IOM and WHO), area of residence was associated with high salt consumption with people in the coastal region consuming more than those living in other areas. Occupation was also associated with high salt consumption: housewives consumed significantly more than people in other occupations. When using the WHO cutoff, results showed that females consumed significantly more salt than males (Table 1).

**Table 1:** Characteristics of the study population by proportion consuming more salt than amount recommended by IOM and WHO

**Characteristics**	**N**	**%**	**Proportion (%) consuming >1.5 g sodium/d (IOM)**	**Proportion (%) consuming >1.96 g sodium/d (WHO)**
*Age (y):*				
25-39	171	44	77.6	65.5
40+	217	56	72.8	57.1
*Sex:*				
Male	198	51	72.2	56.1*
Female	190	49	77.8	66.1
*Occupation:*				
Labour	74	36	72.1	56.4**
Non-labour	140	19	70.3	50.0
Housewives	174	45	78.7	69.0
*Education:*				
0 years	143	37	74.8	60.8
1-5 years	198	51	73.2	59.1
5+ years	47	12	80.9	68.1
*Body Mass index: (kg/m*^*2*^*)*				
Underweight (<18.5)	93	24	68.8	55.9
Normal weight (18.5-24.9)	235	61	75.3	60.9
Overweight (≥25)	56	15	80.4	67.9
*Blood pressure:*^Ɨ^				
Normotensive	353	91	75.1	61.6
Hypertensive	34	9	70.6	52.9
*Geographical distribution:*				
Coastal area	155	40	83.2**	70.3**
Hilly area	111	29	64.0	50.5
Plains area	122	31	73.8	58.2
*Wealth Index:*				
Lowest	106	33	80.2	65.1
Middle	107	34	71.0	57.9
Highest	106	33	72.6	56.6
*Urinary volume (l) Mean(SD)*	1.5(±0.79)			
*Urinary Salt (g) Mean(SD)*	6.8(±4.16)			

**p < 0.01, *p < 0.05 , ^Ɨ^Normotensive: Systolic <140 Hg mm and diastolic <90 Hg mm.

Hypertensive: Systolic ≥140 Hg mm or diastolic ≥90 Hg mm.

According to logistic regression, using the IOM cut off compared to those who lived in hilly areas the odds of high salt consumption were 1.7 times higher among people living in the plains and 3.3 times higher among those living in the coastal area. Compared to people in labour-intensive occupations the odds of high salt consumption were 1.9 times higher among housewives (Table 2). The model using the WHO cut off of 5 g/day yielded similar results (Table 2).

**Table 2:** Correlates of high salt intake based on both IOM (>1.5 g sodium/d) and WHO (>1.96 g sodium/d) recommendations

**Variables**		**Odds ratio (IOM)**	**95% CI**	**Odds ratio (WHO)**	**95% CI**
*Occupation*	Labour	Ref*		Ref**	
	Non-labour	0.95	0.50-1.80	0.80	0.44-1.43
	Housewife	1.90	1.09-3.30	2.21	1.35-3.63
*Geographical area*	Hilly	Ref**		Ref**	
	Plains	1.74	0.98-3.11	1.62	0.95-2.79
	Coastal	3.31	1.81-6.06	3.06	1.78-5.28

*p < 0.05, **p < 0.001.

## Discussion

Our study is the first to describe the level and correlates of salt consumption among adults in coastal Bangladesh. The mean salt consumption of this population was 6.7 g/d (or 115 mmol Na/d) is comparable to those reported in Australia [21], Finland [22], Ghana and West Africa [23] and lower than those reported in other developed countries [24] and in South East Asian countries [6]. Also, the level of salt consumption was less than that reported in a study of pregnant women in the coastal belt of Bangladesh [15]. However, 75% of the study population still consumed more than their biological need and 59% of the population consumed more than the WHO recommended 5 g/d of salt. Having this much of the population exceeding the recommended levels of salt consumption is very concerning given the alarming levels of hypertension present in Bangladeshi adults [25]. Although results from the study area may not be representative of the nation, it is applicable to the coastal area of Bangladesh where 20 million people live [14] and are likely to be exposed to high levels of environmental salinity due to climate change.

Area of residence was by far the strongest predictor of high salt consumption in our study with those in the coastal area consuming the most. This is the first study to indicate that even with areas in close proximity, living directly in coastal areas makes a difference in salt consumption. Globally there is a rising concern that climate change will critically affect the freshwater resources of the world [26]. In Bangladesh, a country bearing some of the worst effects of global climate change, the rivers and ground water in coastal area are threatened by increased salinity from the Bay of Bengal [15]. The coastal population relies heavily on rivers, ground water and ponds for washing, bathing, and drinking water which could result in increased exposure to sodium [27]. Saline intrusion in the groundwater could also affect the sodium content of foods produced, and, therefore, cause increased consumption of salt without people being aware that their intake is increasing. Researchers have reported that excess salinity can be toxic to plants leading to reduced plant yield and plant death [28]. Depending on the variety of plants, sodium can accumulate within different parts of the plant despite existence of good mechanism of excluding sodium within the plant system [28]. It is important to investigate both these indirect sources of salt consumption and the effects of increased salinity on the available food supply.

In our study housewives had significantly higher salt intake than people (males) in labour-intensive occupations. As the majority of the women we studied were housewives, we found that women tended to have higher salt intake then men. The average salt intake of females (7 g) is slightly less than those reported among pregnant women in one coastal location of Bangladesh [15]. However, in studies around the world researchers have shown that women tend to consume less salt than men [6,29]. Researchers have explained that the higher salt consumption among men was due to higher food intake. The explanation for the sex difference in salt consumption in our study may well lie in the context. In our study the men were engaged in labour-intensive occupations whereas women were mostly housewives. As 24 h urinary excretion takes no account of electrolyte loss other than through the kidneys [18], it is possible that the salt intake of men engaged in labour-intensive activities in Bangladesh’s tropical climate may have been underestimated. Another caveat to the explanation could be that housewives had better access to discretionary salt during meal times and, therefore, consumed more. To understand the sex differential in salt consumption it may be important to give further consideration to the context in which salt is consumed.

There have been reports of a growing prevalence of hypertension in Bangladesh [30-32], a trend predicted to continue in coming years. According to a recent Bangladeshi national survey (2011), 19.4% of males and 31.9% of females above 35 years of age were hypertensive [33]. To complicate the scenario, saline intrusions due sea-level rise and other effects of climate change have put 20 million people in the coastal belt at health risk from environmental exposure to sodium [14,34]. Many researchers have shown that reduction of population-level salt intake is a cost-effective public health strategy [35,36]. However, despite the cost-effectiveness, only a few countries have made progress implementing such a public health strategy [36]. For Bangladesh, in the absence of national data on salt consumption, and with existing geographic variations in salt consumption, it is important to understand the different sources of dietary salt and the beliefs and practices around salt use for designing appropriate strategies for different communities. In view of the deleterious health consequences of high salt intake, special attention should be given to coastal areas prone to saline intrusion in food and water sources and areas where cheap raw salt is easily available [34,37]. So far the literature on climate change and health points to the proliferation of infectious disease (diarrhea), vector born disease (malaria) and diseases and health conditions due to migration and loss of livelihood [13]. However, direct health impacts of increased environmental salinity have yet to be addressed adequately. In view of the rising impact of climate change and the large number of people who will be affected, the link between increased salinity and health warrants attention.

### Strengths and limitations

24 hour urine collection was used to estimate salt intake. This is considered the preferred method for the estimation of salt intake but it is not without limitations. One, 24 hour sodium collection may underestimate sodium excretion due to sodium being lost in sweat; and, two, there is no true method to determine under and over collection. We did however determine completeness of the urine samples both by asking each participant and by measuring urine volume. Other methods such as levels of creatinine and PABA to validate urine collection have been used in research elsewhere but were not used in this study. Some studies also used 24 hour dietary recalls to validate urinary sodium. In our study population salt is added to food mostly during preparation and packaged or pre-prepared foods were not eaten that frequently. As there is no published report for nutrient content of mixed Bangladeshi foods we decided not to conduct dietary recall.

The sample size was small and the method of participant selection meant that our participants were those who were available at the time of the home visit; this could have biased the sample. The level of salt consumption reported in the study does not represent the national levels.

## Conclusions

Although the levels of salt consumption in the study population are lower than in some countries, more than three-fourths of the study population consumed more than biologically needed, and half consumed greater than the 5 g of salt daily. As salt consumption is a risk factor for hypertension, it is important that major sources of salt consumption are identified. Interventions to raise awareness about the detrimental health effects of excessive salt use should be designed for coastal communities. Looking to the future, policies that help to reduce salt consumption should be formulated for Bangladesh with special attention to climate-change-vulnerable coastal populations.

## Competing interests

The authors report no conflict of interests.

## Authors’ contribution

SR analyzed the data and drafted the paper; SR, MI, TS, SJ, MAK, SMAH, SH designed and implemented the study and provided intellectual input into the analysis; SMAH, MAL, AKS, FK provided technical expertise, input in interpreting the results; and AB provided overall supervision for design, analysis and drafting of the manuscript. All authors read and approved the final manuscript.

## Pre-publication history

The pre-publication history for this paper can be accessed here:

http://www.biomedcentral.com/1471-2458/14/584/prepub
